# Appropriations for the U. S. A. Medical Museum and Library

**Published:** 1874-01

**Authors:** 


### Appropriations for the U. S. Army Medical Museum and Library.

The appropriation committee in Congress have very unwisely, we think, reported in favor of cutting down the appropriation for the Army Medical Museum and Library from Ten Thousand dollars, the usual amount, to Three Thousand—a sum wholly inadequate to insure the progress of the work already undertaken in those valuable institutions.

We are pleased to learn that our law makers are disposed to economize, but they are moving in the wrong direction when they withhold the necessary funds from the Army Medical Museum. From a small beginning this museum has now grown to be the pride of the medical profession of the United States and the admiration of medical men all over the world. It is without doubt the best collection of its kind ever brought together. The library has become in the short space of time since its conception a magnificent collection of medical works, and under the care of the Surgeon-General and his faithful co-laborers has grown far beyond the expectations of its most sanguine friends. All this has been accomplished by a most careful and economical use of the funds at the disposal of its founders; and now that they are beginning to see the fruits of their labors, and the profession of the United States and the world are about to be benifited by them, it is a most serious disappointment and disadvantage to have the funds so necessary to the continuance of the work withheld. We most earnestly hope that the Appropriation Committee will reconsider their action in this respect and place the usual amount of funds at the disposal of those in charge of the Medical Museum and Library.

At the annual meeting of the Erie County Medical Society, held January 13, 1874, the following resolutions were unanimously adopted:

*Whereas*, We regard the Army Medical Museum and Library of great importance to Medical Science and the Medical profession; therefore,

*Resolved*, That we would most urgently request that the appropriation for the Army Medical Museum and Library be continued at the usual amount of Ten Thousand dollars.

